# A Mass Spectrometry-Machine Learning Approach for Detecting Volatile Organic Compound Emissions for Early Fire Detection

**DOI:** 10.1021/jasms.2c00304

**Published:** 2023-04-20

**Authors:** Sarah Kingsley, Zhaoyi Xu, Brant Jones, Joseph Saleh, Thomas M. Orlando

**Affiliations:** † School of Chemistry and Biochemistry, Georgia Institute of Technology, 901 Atlantic Dr, Atlanta, Georgia 30318, United States; ‡ Guggenheim School of Aerospace Engineering, Georgia Institute of Technology, 270 Ferst Dr, Atlanta, Georgia 30313, United States

## Abstract

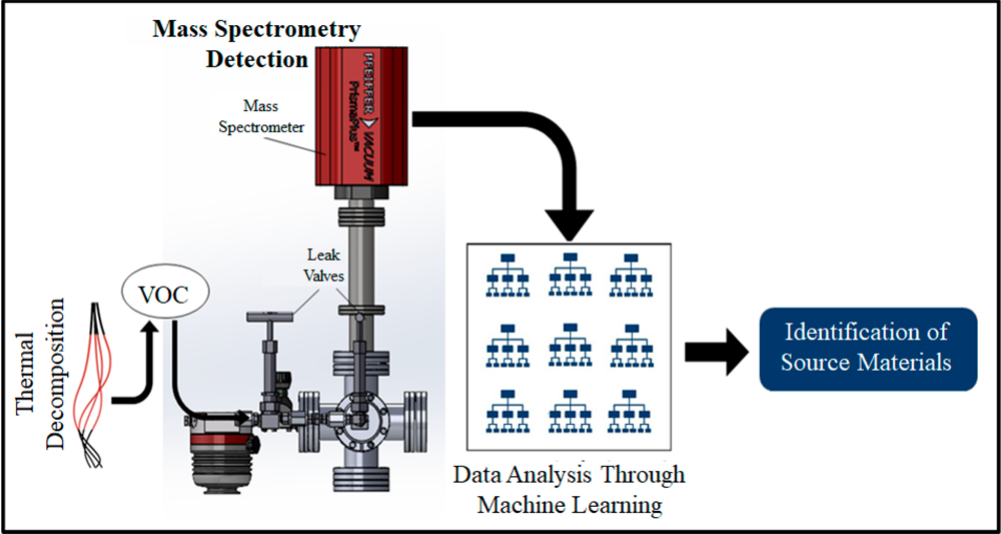

Mass spectrometry in parallel with real-time machine learning techniques were paired in a novel application to detect and identify chemically specific, early indicators of fires and near-fire events involving a set of selected materials: Mylar, Teflon, and poly(methyl methacrylate) (PMMA). The volatile organic compounds emitted during the thermal decomposition of each of the three materials were characterized using a quadrupole mass spectrometer which scanned the 1–200 *m*/*z* range. CO_2_, CH_3_CHO, and C_6_H_6_ were the main volatiles detected during Mylar thermal decomposition, while Teflon’s thermal decomposition yielded CO_2_ and a set of fluorocarbon compounds including CF_4,_ C_2_F_4,_ C_2_F_6_, C_3_F_6,_ CF_2_O, and CF_3_O. PMMA produced CO_2_ and methyl methacrylate (MMA, C_5_H_8_O_2_). The mass spectral peak patterns observed during the thermal decomposition of each material were unique to that material and were therefore useful as chemical signatures. It was also observed that the chemical signatures remained consistent and detectable when multiple materials were heated together. Mass spectra data sets containing the chemical signatures for each material and mixtures were collected and analyzed using a random forest panel machine learning classification. The classification was tested and demonstrated 100% accuracy for single material spectra and an average of 92.3% accuracy for mixed material spectra. This investigation presents a novel technique for the real-time, chemically specific detection of fire related VOCs through mass spectrometry which shows promise as a more rapid and accurate method for detecting fires or near-fire events.

## Introduction

Volatile organic compound (VOC) profiling is a highly informative mass spectrometry (MS) technique that has a number of applications including disease diagnostics, chemical and environmental safety, food quality control, and others.^1−3^ However, the raw output data from VOC analysis is often extensive, and data interpretation can be time-consuming and complicated. While mass spectrometry data of VOCs is typically analyzed through multivariate analysis techniques such as principal component analysis, the use of machine learning in VOC analysis has been shown to provide a more streamlined approach to processing VOC profiling data.^4^

VOC profiling has been demonstrated as a useful detection and identification tool most often in the context of microbial and disease detection.^1−4^ For example, Zhu et al. analyzed the volatile metabolites emitted by four species of bacteria and was able to use principal component analysis (PCA) to differentiate data from each species.^5^ In more recent years, machine learning has been applied to VOC profiling studies. Franchina et al. used gas chromatography (GC) mass spectrometry (MS) to study VOCs emitted by different species of bacteria and then used a random forest to identify chromatographic features distinguishing the volatile profiles of different species.^6^ A similar methodology was used by Stefanuto et al. which analyzed bronchial washings (BALF) and blind bronchial aspirates (BBA) taken from heart and lung transfer patients for markers of pulmonary graft dysfunction (PGD).^7^ VOCs from the BALF and BBA headspaces were analyzed with GCMS, and support vector machine (SVM) was used to identify discriminatory features in the chromatogram that could help to detect PGD.^7^ Arora et al. used a combination of PCA and machine learning to distinguish between fungal and bacterial pathogens based on the VOCs detected with mass spectrometry.^4^ These studies demonstrated the ability of machine learning to identify distinctions in vastly complex data that can be used to accurately classify analyzed samples^1,8,9^

VOC analysis can be performed with a variety of MS instruments. Proton transfer reaction (PTR) MS is of particular interest as it was essentially developed for VOC detection.^10^ PTR-MS uses chemical ionization through a proton transfer reaction between an analyte and a gas within a drift tube. Both the fragmentation pattern and the reaction time aid in identification of analytes, so it is a useful tool when analyzing multicomponent samples. PTR-MS has been used in a variety of VOC applications such as atmospheric chemistry, food science, medical applications, and others. Another popular technique for MS VOC analysis is secondary electrospray ionization high resolution mass spectrometry (SESI-HRMS). SESI-HRMS has high sensitivity and resolution and is able to function in real time without causing harm to the VOC source.^11^ Because of these advantages, SESI-HRMS has been used in a wide range of applications from detecting cancers to analyzing cooking emissions for toxic substances^12,13^

VOC profiling is commonly used in polymer analysis and identification. However, polymers present a challenge for MS analysis because they are difficult to ionize and typically have high molecular weight and low solubility.^14,15^ Certain MS techniques have been used to overcome these challenges such as matrix assisted laser desorption ionization (MALDI) or atmospheric pressure solids analysis probe (ASAP) MS. ASAP-MS has been shown to be an especially effective method for polymer analysis capable of ionizing high molecular weight polymers at atmospheric pressure in the solid state so that dissolving the polymer is unnecessary.^15^

Thermal decomposition-based techniques such as pyrolysis (Py) MS, Py-GC-MS, or thermal desorption and pyrolysis direct analysis in real time (TDPy DART) MS are often used in polymer characterization because these techniques are time-efficient and demonstrate sufficient analyte recovery.^16,17^ Thermal decomposition refers to the process by which molecules break down and produce smaller molecular fragments when exposed to intense heat.^18^ Polymer-based materials are especially susceptible to thermal decomposition processes^18,19^ and can produce a complicated distribution of VOC. Because these volatiles are formed from the original polymer chain, the volatiles may give some indication of the original structure of the molecule. In fact, many studies have demonstrated that the products of thermal decomposition reactions are consistent and, often, unique to the polymer.^14,20−24^ Other research has shown that changes in polymer structure as well as additives can also be detected and studied through thermal decomposition analyses.^14^

This study implements relatively simple MS instrumentation: electron impact mass spectrometry (EI-MS) to analyze the VOCs released through the thermal decomposition of polymers. Traditionally, EI-MS has not been considered appropriate for VOC analysis in part because the EI results in extensive fragmentation which makes analyte identification difficult for complex, multianalyte samples.^10^ To overcome this limitation, this method uses machine learning to process data which is capable of analyzing and classifying complex spectra automatically.

Volatiles are often emitted at lower temperatures than smoke, which is a mixture of volatiles and solid aerosols.^19^ Thus, these VOC products can be useful early indicators of overheating or prefire conditions. As an example, early detection of fire or near-fire events during onboard monitoring of a spacecraft is necessary since spacecraft are confined environments with limited evacuation and resupply options as well as limited ways to extinguish a fire. Fires must be detected as early as possible to mitigate the harm done to the crew and equipment.^25,26^ On board the International Space Station (ISS), the current method of fire detection is the Fire Detection and Suppression system (FDS) which uses an optics-based particle monitor to detect aerosols in smoke.^27^ However, FDS lacks specificity and has a pattern of producing false alarms during housekeeping tasks that increase the amount of dust in cabin air. Lack of specificity is a fundamental problem with the current system and raises concerns for lunar missions where lunar dust has the potential to trigger false alarms in the fire detection system as well.^28^

Chemically specific fire detection is not only applicable to spacecraft but also to many Earth environments. In 2020, structure fires in the United States resulted in 8.7 billion dollars in direct property damage, 11,900 injuries, and 2,630 civilian deaths.^29^ Early detection of fires can help to lessen the damage caused, while chemically specific information about the fire can help effectively extinguish or prevent the fire.^19−24^

This study’s approach to improving fire detection specificity is to develop a method for detecting chemically specific indicators of fires or near fire events using mass spectrometry and machine learning. The target analytes for this method are the volatiles produced in the thermal decomposition of common spacecraft materials. The collected data was used to develop a machine learning classification capable of determining the material or materials that produced the VOCs detected with high accuracy and very limited data preprocessing.

## Experimental Section

### Tested Materials

Three materials commonly used on spacecraft were selected for this study: biaxially oriented polyethylene terephthalate (Mylar), poly(methyl methacrylate) (PMMA), and polytetrafluoroethylene (Teflon). Teflon is used in spacesuits, cargo liners, wire insulation, and sampling bags, while PMMA and Mylar make up the windows and thermal insulation, respectively.^30^ Each material was purchased from McMaster Carr and used as is. Teflon was purchased in the form of Teflon tape, and Mylar and PMMA were purchased in sheets. Samples used in single material runs had an approximate volume of 1.2 cm^3^. Mixtures containing two materials were prepared to be 50% by volume of each of the two materials, and mixtures of all three materials were comprised of 33% by volume of each material. Each material’s density was used to calculate the mass of that material needed for each run. Raw materials were cut into smaller pieces which were weighed out to the necessary mass using a VeriTas Analytical Balance.

### Sample Analysis

**Figure 1:**
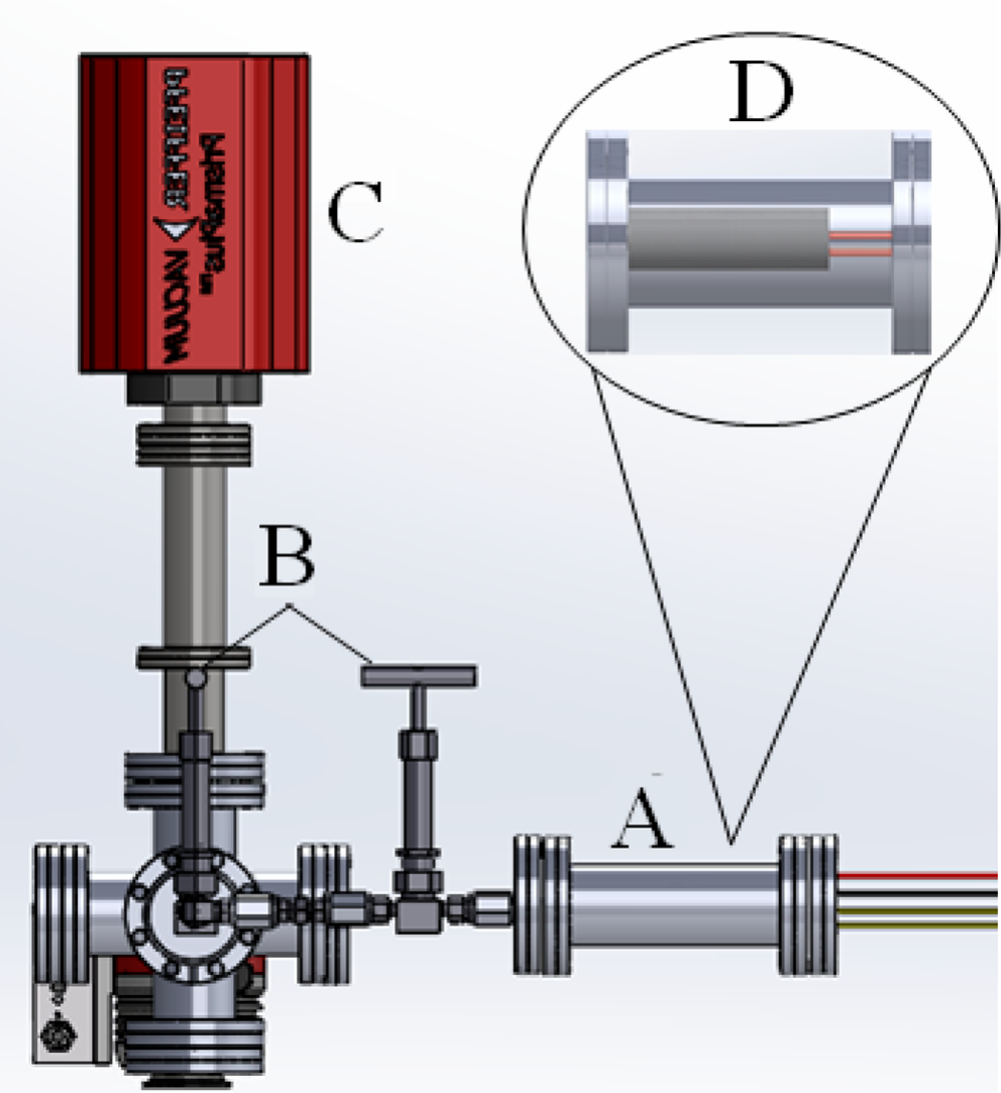
Mass spectrometry system for analysis of thermal decomposition products. (A) Sampling tube where samples are heated using the sample heater shown in (D). (B) Leak valves used to dose volatiles into the portion of the chamber that is kept under vacuum. The EI-QMS used to analyze the VOC produced in the sampling tube is shown in (C).

The sampling and analysis instrumentation used in this study is shown in Figure 1. This study utilized a differentially pumped, quadrupole mass spectrometry system with an attached sample heating unit. The sample heater shown in Figure 1A,D was constructed using 80:20 Ni/Cr wire with a resistance of 4.094 ohms/ft coiled around a ceramic tube. Alumina oxide insulation was wrapped around the sample heater to minimize heat lost to the surroundings. An AC voltage source was used to run a current through the coil and heat the sample. The heating rate was controlled by varying the voltage output of the AC voltage source. These heating rates varied from approximately 5–10 °C/min. Samples of each material or a mixture of materials were placed inside the ceramic tube of the sample heater and were then heated from room temperature to approximately 700 °C. A thermocouple temperature probe was mounted inside the ceramic tube in contact with the sample to measure the sample temperature over the course of the run.

Sample heating took place under typical atmospheric conditions: The pressure in the sample chamber was kept at 760 Torr and samples were exposed to lab air consisting of 78% N_2_ 21% O_2_. These conditions also reflect the environmental conditions on board spacecraft.^31,32^

Volatiles were directed into the chamber through the differentially pumped leak valves shown in Figure 1B. Differential pumping maintained the pressure differential between the sample heater chamber and the MS chamber equipped with a PrismaPlus Quadrupole Mass Spectrometer (QMS) which can be seen in Figure 1C. The QMS was used to collect data on the VOCs emanating from the samples upon heating. The QMS instrument has a mass range of 1–200 *m*/*z* and a limit of detection (LOD) on the order of 10^–13^ Torr inside the MS chamber or approximately 400 ppm at atmospheric pressure. This sensitivity was achieved using a faraday detector. The QMS is also equipped with an SEM detector which is expected to improve sensitivity and achieve an LOD of approximately 1 ppm.

Prior to analysis of the selected materials, extensive background measurements were performed. Spectra of the background with and without each piece of sampling equipment were collected. Additionally, background spectra were collected of the sampling system with no sample at temperatures ranging from room temperature to 700 °C to assess any volatile contributions from the instrument.

### Data Collection and Analysis

**Figure 2:**
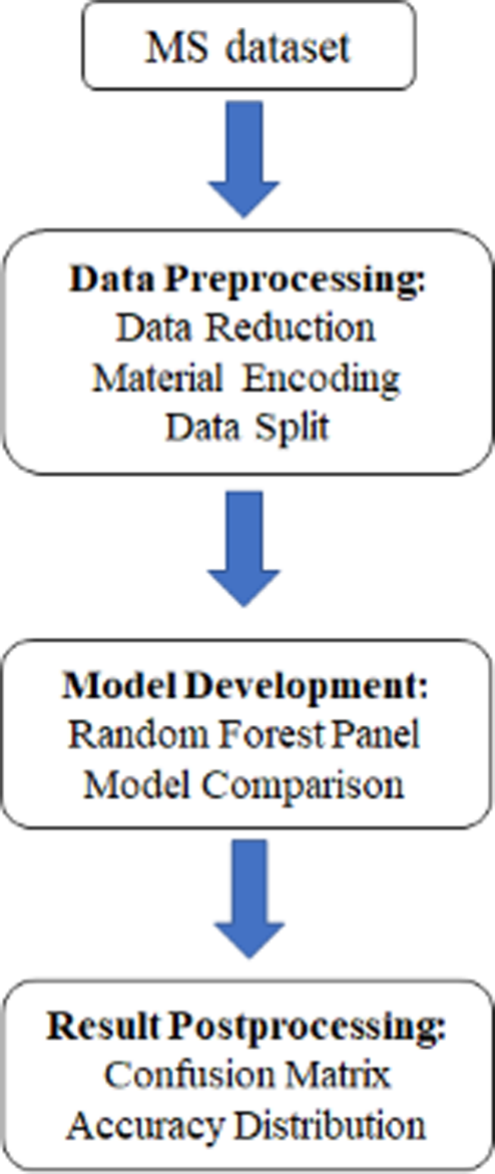
Machine learning workflow used to develop and evaluate the classification used in this study. Data sets of mass spectra were processed and split into training and testing sets. Next several machine learning techniques were used to classify this data, including a random forest panel. The accuracy of each technique was calculated and compared. The random forest panel achieved the highest accuracy and was therefore the selected technique for this investigation.

As shown in Figure 2, the data collection and machine learning classification development involved four main steps: (1) MS data set development, (2) data preprocessing, (3) model development and accuracy testing, and (4) postprocessing of the results. Each step will be described in more detail below:

#### MS Data Set Collection

Mass spectra were collected on each material in full scan mode so that each mass within the 1–200 *m*/*z* mass range was scanned at an interval of 0.03 *m*/*z*. Samples were heated from room temperature to up to 700 °C, and spectra were collected throughout the heating process. Heating ramp rates varied from approximately 5 to 10 °C/min.

Detected thermal decomposition products were identified to help ensure that the chemical signature for each material was consistent over each run and different from the chemical signatures for other materials studied. Product identification also helped to ensure that detected products were consistent with the literature on the studied materials. The products identified in this investigation are the expected products based on the information available. However, for the purpose of machine learning it is not necessary to identify the molecular formula of individual peaks. As long as the spectral pattern serving as the chemical signature is consistent and unique for each material, the machine learning model will be able to perform the classification.

To perform the product identification, full scan data was collected to determine what peaks were detected during the analysis of each material. Data was then collected in Multiple Ion Detection (MID) mode by selecting the masses of detected peaks and tracking the growth and decay of certain masses over time. If two masses exhibited the same pattern of growth and decay, it was concluded that they were molecular fragments of the same molecule. These analyses made it possible to identify groups of peaks related to different products. To identify the products detected, spectra of the products from selected materials were acquired through the National Institute of Standards and Technology database.^2,20,22,33−36^ The NIST spectra were examined to determine if any reported products for a given material had peaks in common with the collected spectra for that material. For each material, products were identified that had the same peaks as those detected in the spectra collected for this study.

Distribution of peak abundance was also used to verify if a product had been detected. The relative abundances of each peak in the NIST spectra were compared to the data collected in this study. Specifically, the distribution of peak abundances for all of the peaks of a single product were examined to determine if the distribution of abundances in the NIST spectra matched the distribution of abundances in the collected spectra.

The temperatures at which each material began producing thermal decomposition products were also determined by taking measurements in MID mode. A selected subset of detected masses for a given material were monitored. The partial pressure of each mass was tracked over time at an interval of 100 ms. The temperature of the sample was also measured every second over the course of the run. Time of detection was correlated with sample temperature to determine the temperature range for which products were first detected. The MID data served only to aid in determining which peaks were relevant to the thermal decomposition and in determining the temperature range at which products were first detectable. Only full scan data was included in the data sets used to train and test the machine learning model.

#### Data Preprocessing

Data sets of full scan spectra for each material, Mylar, Teflon, and PMMA, and each mixture of material, Mylar-Teflon, Mylar-PMMA, Teflon-PMMA, and Mylar-PMMA-Teflon, were compiled. Each spectrum was treated as an individual data point and processed as follows to prepare the data for the machine learning classification.

The QMS full scan mode recorded the partial pressure at each *m*/*z* at an interval of 0.03 *m*/*z*. A data reduction method to condense the number of features in each data set was used. Specifically, the raw output data was reduced so that only integer and half integer *m*/*z* values within the mass range of 1–200 *m*/*z* were kept in the input data for the machine learning model. Data reduction was performed using a MATLAB script that expressed all peaks as Gaussians centered on the integer value.

Since an encoded MS data set is more suitable for the machine learning classification, the original categorical MS data was converted into both a linear and binary code. Linear encoding was used to assign values 0–6 to each class of material and binary encoding enabled the classification of mixtures based on the popular One-Hot encoding concept in machine learning.^37^ The linear and binary encoding results for different materials and mixtures are shown in Table 1.

**Table 1:** 

Types	Material	Linear code	Binary code
Single	Mylar	0	(1, 0, 0)
	Teflon	1	(0, 1, 0)
	PMMA	2	(0, 0, 1)
Mixture	Mylar-Teflon	3	(1, 1, 0)
	Mylar-PMMA	4	(1, 0, 1)
	Teflon-PMMA	5	(0, 1, 1)
	Mylar-Teflon-PMMA	6	(1, 1, 1)

The encoded MS data set was split into training and testing subsets by randomly selecting 80% of the single material data (Mylar, Teflon, and PMMA) as the training subset.^38^ The entire MS data set including the three single materials (Mylar, Teflon, and PMMA) and the four mixtures (Mylar-Teflon, Mylar-PMMA, Teflon-PMMA, and Mylar-Teflon-PMMA) was used as the testing data set.

#### Model Development

After establishing mass spectra data sets consisting of full scan spectra, the machine learning workflow initially involved the development of a random forest panel (RFP) classification model based on the binary encoded MS data set. A random forest classifier is characterized by two distinctive aspects: random sampling and random feature selection, which are used to decorrelate the different trees in the forest and thus lower the variance of the prediction to avoid overfitting.^39^ The RFP was trained with the training subset. The trained RFP classification accuracy was assessed using both single material and mixed material data. The accuracy was then compared with other advanced machine learning classifications, including K-nearest neighbors (KNN),^40^ decision tree (DT),^41^ support vector classification (SVC),^42^ Naïve Bayes (NB),^40^ and multilayer perceptron (MLP).^43^

**Figure 3:**
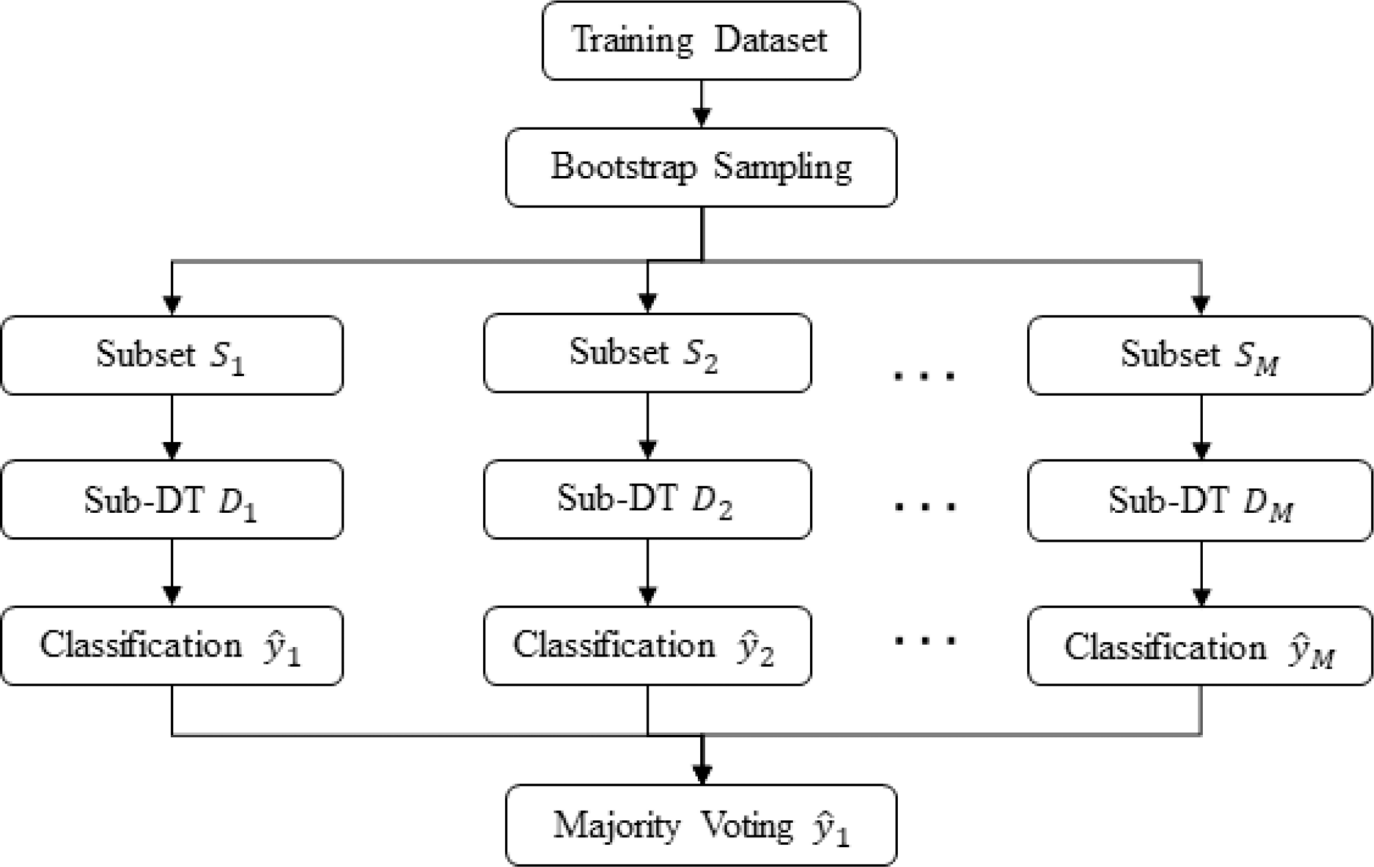
Random forest training process showing how data is split into subsets and processed through decision trees which each provide a classification. The overall classification is the class that the majority of trees assigned the data point to.

All the steps in the RFP training process are shown in Figure 3. First, the training data set is split into M subsets by the Bootstrap method, where M is the hyperparameter indicating the number of sub-DTs. Second, M individual sub-DTs are “planted”, and each provides a classification prediction, *ŷ*_*m*_. Finally, an overall prediction, *ŷ*, is achieved through a majority voting from each individual prediction.

**Figure 4:**
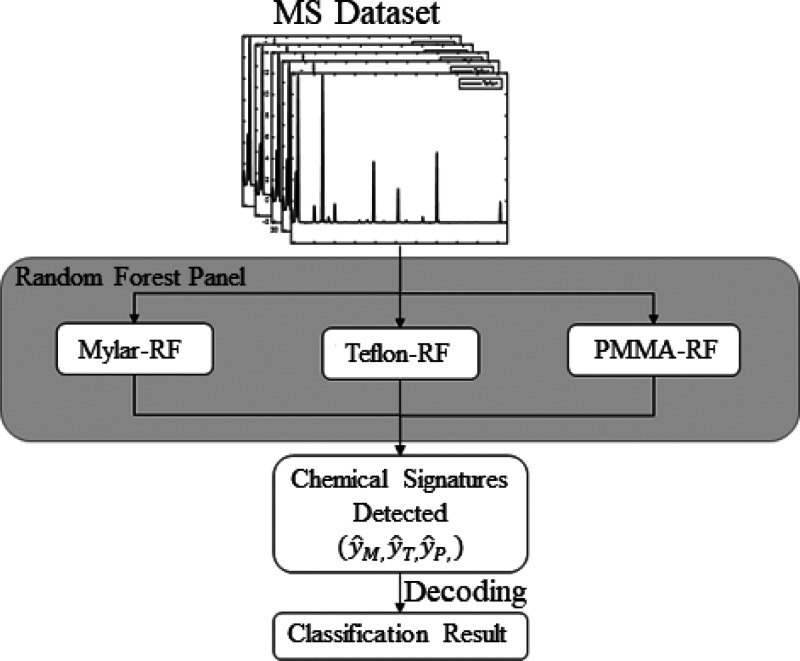
Full random forest panel workflow where all mass spectra in the prepared data set are processed through three random forest classifiers and a classification result is assigned.

The trained RF with sub-DTs was then used to predict the label of the spectra in the testing data set. Based on the RF, a RFP containing three sub-RF models, Mylar-RF, Teflon-RF, and PMMA-RF, is shown in Figure 4. First, the mass spectra were input independently into the three sub-RFs. The output of the sub-RFs is the classification results for the corresponding material. For example, if the Mylar-RF output, *ŷ*_*M*_ = 1, it indicates Mylar is detected in the environment. Otherwise, *ŷ*_*M*_ = 0 means the RFP does not detect Mylar in the environment. Second, the classification results from different sub-RFs were summarized to achieve binary classification results (*ŷ*_*M*_*, ŷ*_*T*_*, ŷ*_*P*_). Finally, the binary classification result was decoded based on Table 1 to indicate the final result. For example, if the Mylar-RF output *ŷ*_*M*_ = 1, Teflon-RF output *ŷ*_*T*_ = 0 and PMMA-RF output *ŷ*_*P*_ = 1, then the binary classification result is (1, 0, 1). The binary results were then decoded as listed in Table 1, with the corresponding linear code of 4 for the Mylar–PMMA mixture. The training methodology allowed for flexibility in the RFP by allowing the user to simply add and train additional sub-RFs for other materials to expand the classification capability of the RFP for more materials without rebuilding and retraining the entire model.

#### Result Postprocessing

Postprocessing to comprehensively analyze the RFP classification results for different materials was also conducted. The classification results were first visualized with a confusion matrix. A confusion matrix is an *n* × *n* matrix used for evaluating the performance of a classification model, where *n* is the number of target classes (*n* = 7 from Table 1). The matrix compares the actual target values with those predicted by the RFP model. This gives a holistic view of the accuracy of the classification model and the error types of incorrect predictions.^44,45^ A confusion matrix has two dimensions; one dimension is indexed by the true label of an object, and the other is indexed by the class that the model predicts.

**Figure 5:**
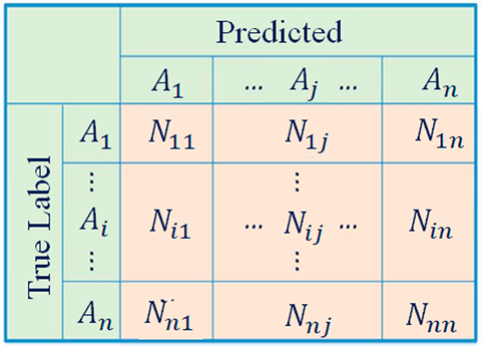
An example of the confusion matrix of classes *A*_1_, *A*_2_, ..., *A*_*n*_.^44^

Figure 5 shows the basic form of the confusion matrix for multiclass classification, with the classes *A*_1_, *A*_2_, ..., *A*_*n*_. In the confusion matrix, *N*_*ij*_ represents the number of samples of class *A*_*i*_ classified as class *A*_*j*_. Based on the confusion matrix results, an accuracy distribution to show the classification results of each category more straightforwardly was obtained. The accuracy distribution of material *i* (*i* = 0, 1, 2, ..., 6) is calculated by eq 1, and the average accuracy of all material categories is calculated by eq 2.
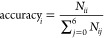
1
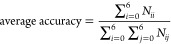
2

## Results and Discussion

### Identification of Fire-Related Chemical Signatures

**Figure 6:**
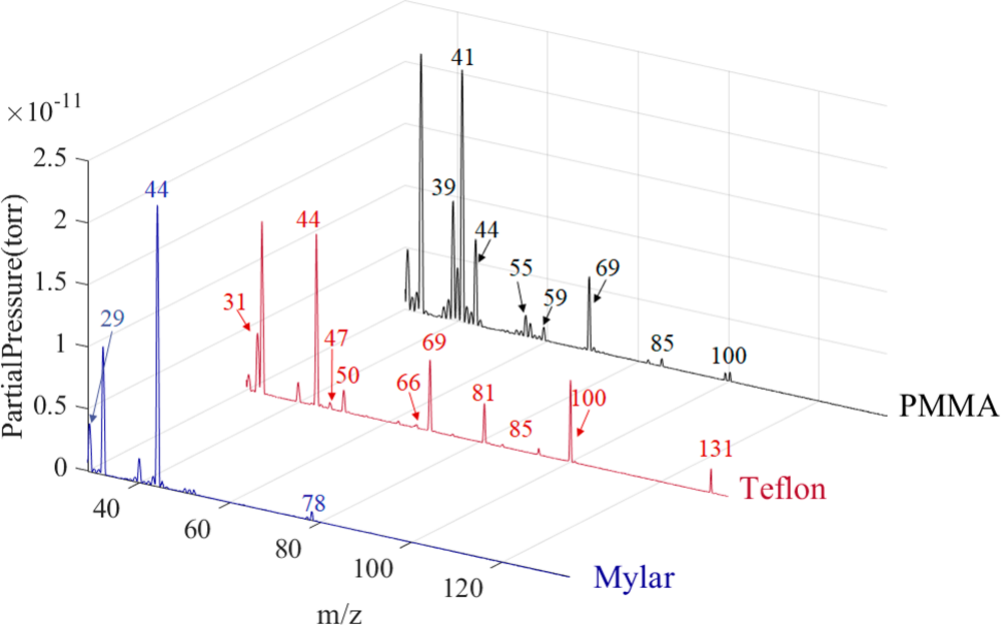
Mass spectra above represent the spectral patterns that serve as chemical signatures for each material in the RFP classification. The PMMA spectra shown were collected at 484 °C, while the Mylar spectra were taken at 467 °C, and the Teflon spectra were taken at 470 °C. The data was collected in the presence of ambient air, so oxygen, nitrogen, and other atmospheric gases were detected in the spectra. The 29–135 *m*/*z* region shown in this figure exhibits the most material-specific peaks while excluding most peaks caused by air which are more abundant below 29 *m*/*z*.

The measured composite mass spectra for Mylar, Teflon, and PMMA are shown in Figure 6 and described in more detail below. Mass spectra scans were obtained as a function of temperature which allowed for the identification of temperatures at which thermal decomposition products were first detectable. The mass spectra in Figure 6 were taken in the 450–500 °C range and show multiple well-resolved, reproducible peaks with Mylar yielding the least complex spectra. Spectra were collected in the presence of air, so only the 29–135 *m*/*z* region is shown in Figure 6 as most peaks below this mass range are due to air. All observed/identified reaction products for each material studied are reported in Table 2, as well as the temperature at which each product was first detected.

**Table 2:** 

Material	Detected Products		Identifying Masses (*m*/*z*)	Onset Temperature Range (°C)	Smoke Onset Temperature Range (°C)
**Mylar**	Carbon Dioxide	CO_2_	44	300–325	390–400
	Acetaldehyde	CH_3_CHO	29, 44	300–325	
	Benzene	C_6_H_6_	78	325–375	
**PMMA**	Carbon Dioxide	CO_2_	44	200–250	300–330
	Methyl Methacrylate (MMA)	C_5_H_8_O_2_	39, 41, 55, 59, 69, 85, 100	200–250	
**Teflon**	Carbon Dioxide	CO_2_	44	440–460	495–500
	Tetrafluoromethane	CF_4_	31, 50, 69	440–460	
	Tetrafluoroethene	C_2_ F_4_	31, 50, 81, 100	440–460	
	Hexafluoroethane	C_2_F_6_	31, 50, 69	440–460	
	Hexafluoropropene	C_3_F_6_	31, 69, 81, 100, 131	440–460	
	Carbonic difluoride	CF_2_O	47, 66	450–470	
	CF_3_O	CF_3_O	85	450–470	

**Figure 7:**
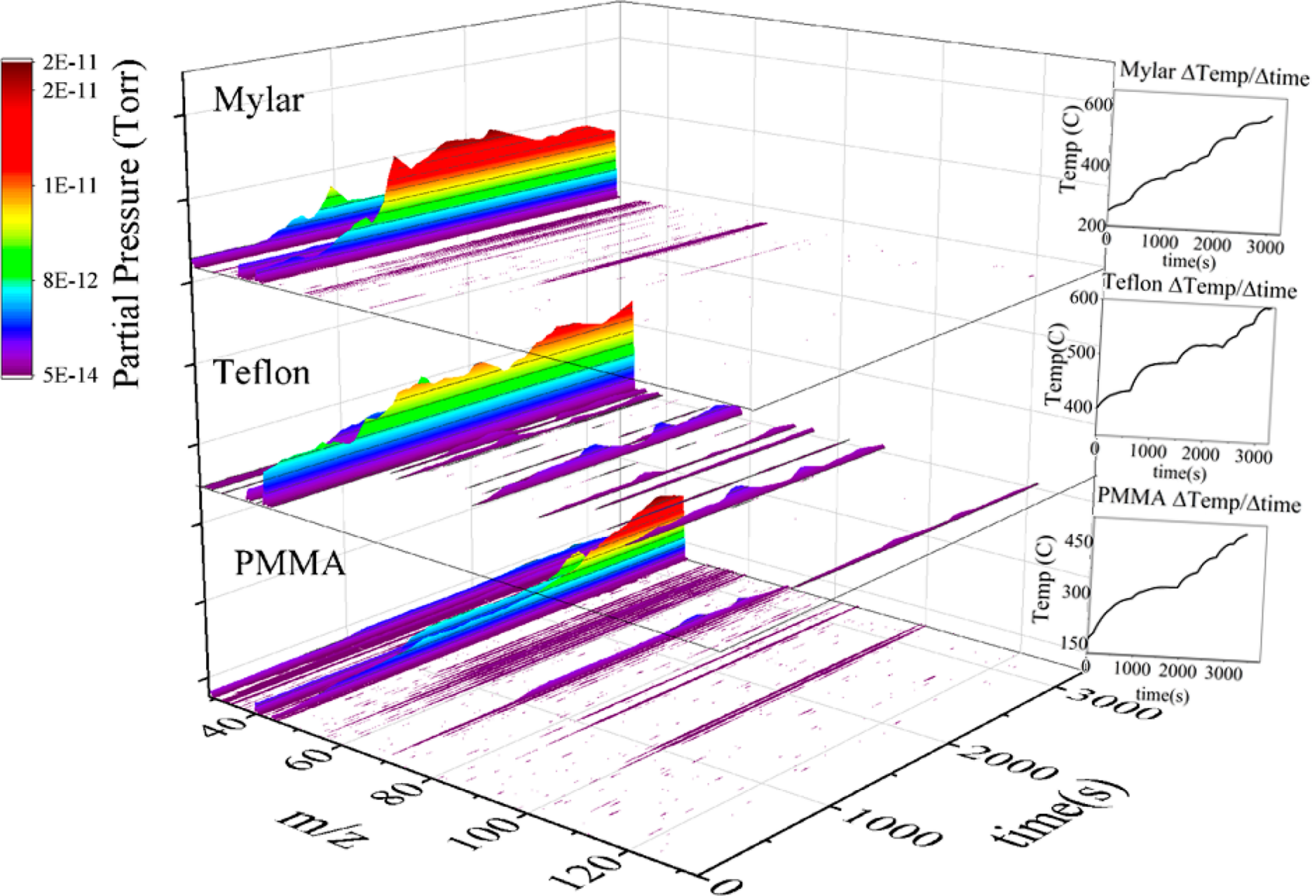
Surface plot created by compiling all full scan data collected during a single run of Mylar (top), Teflon (middle), and PMMA (bottom). The masses of the detect peaks are shown along the *x*-axis, reaction time is shown on the *y*-axis, and peak intensity expressed in partial pressure is shown on the *z*-axis according to the color bar to the left of the plot. The change in temperature over time is shown on the right-hand side of each plot.

Compilations of all full scan spectra collected over single runs of Mylar, Teflon, and PMMA are shown in Figure 7. The stacked surface plots show the growth and decay of all products as a function of time. The spectra are related to the change in temperature over time through the temperature plot on the right-hand side of each surface plot.

Multiple runs yielded similar results with slight variations in absolute intensities as is typical for mass spectra scans. The mass resolution is at least unit mass, and the positions do not change. It is also clear that each species from each sample has a different temperature dependence which is critical information to capture for prognostic detection.

### Mylar

Based on the spectra shown in Figure 6, detected thermal decomposition products during analysis of Mylar included carbon dioxide (CO_2,_ 44 *m*/*z*), acetaldehyde (CH_3_CHO, 29 and 44 *m*/*z*), and benzene (C_6_H_6,_ 78 *m*/*z*). CH_3_CHO and CO_2_were detected between 300 and 350 °C, whereas C_6_H_6_ was detected in the range of 325–375 °C. The onset of these products can be seen in the top panel of Figure 7. During the Mylar thermal decomposition reactions, smoke was observed at 390–400 °C. This temperature of smoke onset gives this system a temperature window where products were detected but no smoke was observed.

### Teflon

Multiple volatile molecules were also observed as products of Teflon’s thermal decomposition reactions. The onset and growth of the detected products can be seen in the middle panel of Figure 7. The primary peaks were identified as fluorocarbons: tetrafluoromethane (CF_4_, 31, 50, and 69 *m*/*z*), tetrafluoroethene (C_2_F_4_, 31, 50, 81, and 100 *m*/*z*), hexafluoroethane (C_2_F_6_, 31, 60, and 69 *m*/*z*), and hexafluoropropene (C_3_F_6_, 31, 69, 81, 100, and 131 *m*/*z*), two fluorocarbons with bound oxygen: carbonic difluoride (CF_2_O, 47, and 66 *m*/*z*) and CF_3_O (85 *m*/*z*), and CO_2_. The Teflon fluorocarbon products and carbon dioxide were detected between 440 and 460 °C. The other products, CF_2_O and CF_3_O, were detected at slightly higher temperatures ranging from 450 to 470 °C. Smoke onset was observed at a higher temperature range of 495–500 °C. Overall, Teflon was stable at higher temperatures than Mylar and, as discussed below, PMMA.

### PMMA

Methyl methacrylate (C_5_H_8_O_2_, 39, 41, 55, 59, 69, 85, and 100 *m*/*z*) and CO_2_ (44 *m*/*z*) were identified as primary thermal decomposition products from PMMA. All peaks shown in the PMMA spectra in Figure 6 and the bottom panel of Figure 7 represent fragments of C_5_H_8_O_2_ except for the peak at 44 *m*/*z* which represents CO_2._ However, only the most abundant masses that were most useful in identifying this product are listed in Table 2. The numerous fragments produced by C_5_H_8_O_2_ during MS analysis allowed a distinct chemical signature to be established even though only two products were detected. Both C_5_H_8_O_2_ and CO_2_ were detected between 200 and 250 °C while smoke was not observed until the material reached 300–330 °C. Thus, PMMA exhibited the greatest difference in the temperature at which thermal decomposition products were first detected and the temperature at which smoke was observed.

### Mixed Samples

**Figure 8:**
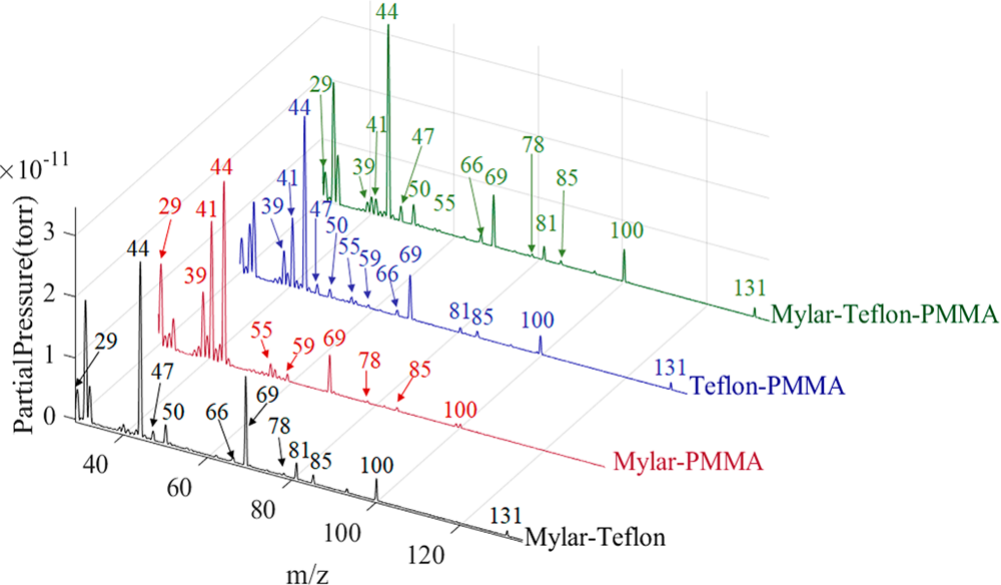
Top spectra show data collected during the thermal decomposition of mixed samples. The spectra were collected at the following temperatures: Mylar-Teflon: 538 °C, Mylar-PMMA: 404 °C, Teflon-PMMA: 525 °C, Mylar-Teflon-PMMA: 525 °C. The chemical signatures for each material shown in Figure 6 are detectable in the mixed spectra as well.

Mixed samples containing either two or all three materials were also studied. MID and full scan data was collected for each mixture, and examples of the full scan spectra can be seen in Figure 8. This mixed material study is important since it is necessary to establish credibility for any prognostic monitoring and analysis system. The data shows that the mixed sample produced the same products as the individual samples and no reaction products. The product onset temperatures were also consistent between the mixed and individual samples. In addition, peak ratios for peaks found in the spectra of only one individual material remained consistent between individual and mixed spectra. However, Teflon and PMMA both had peaks at 69, 85, and 100 *m*/*z*. The peaks at 69 and 85 *m*/*z* represent molecular fragments of C_5_H_8_O_2_, and the peak at 100 *m*/*z* represents the molecular ion in the PMMA spectra. However, in the Teflon spectra the peak at 69 *m*/*z* is due to fragments of C_2_F_4_, C_2_F_6_, and C_3_F_6_. The peak at 100 *m*/*z* is due to fragments of tetrafluoroethene and C_3_F_6_, and the peak at 85 *m*/*z* is CF_3_O. Spectra taken of mixtures containing both Teflon and PMMA showed increases in these peaks since both materials contributed to their abundance.

### Machine Learning Classification

**Figure 9:**
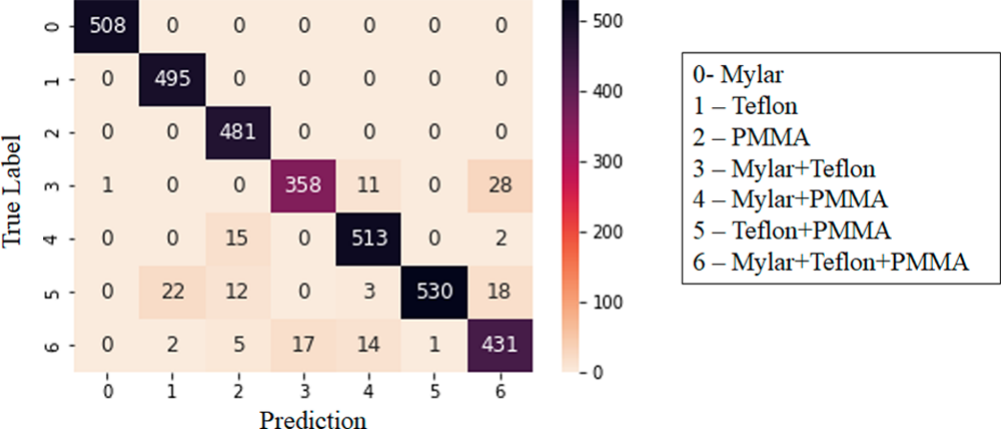
Confusion matrix showing results of the random forest panel (RFP) classification. The true label of each piece of data included in the testing data set is indicated by the row number while the prediction or the label assigned by the RFP is indicated by the column number. Diagonal elements show the number of spectra that were correctly classified (where the true label matches the prediction). Off-diagonal elements show the number of misclassified spectra.

Using the data and machine-learning approach described above, the results of the RFP demonstrated strong accuracy in the classification of both single material spectra and mixed material spectra. In Table 3, the classification accuracy distribution results show 100% accuracy for the classification of single material data. Mixed data classification accuracy ranged from 90% to 96.8% with an average of 92.3%. The confusion matrix shown in Figure 9 is diagonally dominant so that *N*_*ii*_ ≫ *N*_*ij*_ (*i* ≠ *j*). The minimum number of diagonal elements is *N*_3,3_ = 358, which is more than ten times greater than the maximum of the off-diagonal element, *N*_3,6_ = 28. This indicates that the trained RFP can achieve perfect prediction accuracy for data of a single material and a high level of prediction accuracy for mixed material data.

**Table 3:** 

Types	Material	Linear code	Binary code	Classification Accuracy (%)
Single	Mylar	0	(1, 0, 0)	100.0
	Teflon	1	(0, 1, 0)	100.0
	PMMA	2	(0, 0, 1)	100.0
Average:				100.0
Mixture	Mylar-Teflon	3	(1, 1, 0)	90.0
	Mylar-PMMA	4	(1, 0, 1)	96.8
	Teflon-PMMA	5	(0, 1, 1)	90.6
	Mylar-Teflon-PMMA	6	(1, 1, 1)	91.7
Average:				92.3

The confusion matrix and accuracy distribution show the maximum prediction accuracy for mixed materials is 96.8%. This level of accuracy was achieved for Mylar–PMMA: 4, and the minimum accuracy is 90.0% for Mylar–Teflon: 3. The misclassified data included both false positives and false negatives, so in some cases, chemical signatures that were present in the spectra were not detected. In other cases, signatures that were not present in the spectra were detected. The randomness in the misclassifications and the small variance of prediction accuracy of mixtures indicates the trained RFP prediction is unbiased and free from overfitting.^46,47^ The superior accuracy for the single material data classification is to be expected because the model was trained using only single material data. Therefore, the model performs best on data that most resembles the training set. Mixed data was not included in the training set to enable a more flexible classification architecture to which new materials can be added. However, excluding mixed data from the training set also caused a drop in the accuracy in the mixed data classifications. The overall accuracy of the RFP including the mixed classifications is high, so these results indicate that the RFP can accurately predict and classify data on mixtures of materials with the training of single material data. The use of the RFP allows this method to (1) specifically detect VOCs indicating a fire or near fire event and (2) extract further information about the event by identifying a material or materials involved in that event. The flexible framework of the model allows for the addition of new materials, so the model is adaptable to the monitored environment.

### Potential Spacecraft Application

The volatiles detected during the analysis of PMMA and Teflon were distinctive and thus provided a highly specific indicator of a fire or near-fire event involving these materials. However, the major components of the Mylar chemical signature, acetaldehyde, benzene, and CO_2_, have other environmental sources in spacecraft. Acetaldehyde and benzene are both produced through off gassing of materials and human metabolism, and CO_2_ is produced through human respiration.^48,49^ Off gassing of materials contributes approximately 2.5 × 10^–5^ and 1.1 × 10^–4^ mg/day per kilogram of material of benzene and acetaldehyde, respectively, while human metabolism produces 2.2 and 0.6 mg/day per person of benzene and acetaldehyde, respectively.^49^ Human respiration typically generates 1 kg of CO_2_ per person per day.^50^ The Spacecraft Maximum Allowable Concentrations (SMAC) for CO_2_ is 13000 mg/m^3^; however, the Carbon Dioxide Removal Assembly (CDRA) typically maintains a concentration of 2–5 mg/m^3 ^^51^ and proposed systems aim to minimize daily CO_2_ concentrations further.^50^ The SMAC for a 24 h period is 3 mg/m^3^ for benzene and 6 mg/m^3^; however, typical concentrations of these contaminants remain very low, <0.05 mg/m^3^ for benzene and 0.05–0.33 mg/m^3^ for acetaldehyde.

Both benzene and acetaldehyde are scrubbed from spacecraft air using the Trace Contaminant Control (TCC) while CO_2_ concentrations are controlled using CDRA.^49,50^ Because all these contaminants have other sources in spacecraft air it would be necessary to compare chemical signatures to nominal cabin air in order to prevent false alarms. It is expected that a fire or near-fire event would produce increased concentrations of these volatiles compared to nominal conditions. While faults in the TCC could lead to elevated levels of acetaldehyde and benzene and a fault in CDRA could lead elevated CO_2_, simultaneous faults in both systems would be required to mimic the conditions of a fire or near-fire event involving Mylar. As it is unlikely that faults in these systems would occur at the same time, a false alarm due to other environmental sources of acetaldehyde, benzene, and CO_2_ is also unlikely.

A limitation of this project is that it is expected that TCC and CDRA would remove contaminants produced by a fire or near-fire event to some extent. Further testing would be required to determine how volatile scrubbing systems would affect the chemical signature before this method could be applied in a spacecraft environment.

## Conclusions

A mass spectrometry-machine learning method for the identification of VOCs emitted during the heating and eventual combustion of common materials such as Mylar, Teflon, and PMMA has been designed and tested. To enable chemically specific detection of fire and near-fire events involving these materials, VOC signatures were measured during controlled heating, and machine learning was used to reliably identify each material. Data sets of the chemical signatures for each material and mixture of materials were generated to develop an RFP machine learning classification that allowed accurate identification of the source materials and mixtures before combustion. The model achieved a minimum accuracy of 90% in its classification of mixed material spectra and 100% accuracy in the classification of single material spectra. With appropriate MS data sets, the structure of the RFP should allow the classifier to identify VOC signatures in mixtures with unknown contaminants.

The reported results show that this method can provide high accuracy and specificity in the classification of early indicators of fires and near fire events allowing for both the early detection of a fire and the identification of the materials involved in the fire. The early detection and automated data processing capabilities demonstrated in this work are key for advancing fire detection techniques and allowing for more autonomous control of enclosed environments both on Earth and in space.

The implementation of machine learning allowed for the use of simplified instrumentation but still achieved accurate results. The method shows promise as a chemically specific fire detection method and demonstrates how machine-learning techniques can simplify and streamline MS data analysis to facilitate high accuracy VOC profiling and identification.
